# Malaria gametocytogenesis

**DOI:** 10.1016/j.molbiopara.2010.03.019

**Published:** 2010-08

**Authors:** David A. Baker

**Affiliations:** Department of Infectious and Tropical Diseases, London School of Hygiene & Tropical Medicine, Keppel Street, London WC1E 7HT, UK

**Keywords:** Malaria, *Plasmodium*, Gametocyte, Parasite, Drug, Differentiation

## Abstract

Male and female gametocytes are the components of the malaria parasite life cycle which are taken up from an infected host bloodstream by mosquitoes and thus mediate disease transmission. These gamete precursors are morphologically and functionally quite distinct from their asexual blood stage counterparts and this is reflected in their distinct patterns of gene expression, cellular development and metabolism. Recent transcriptome, proteome and reverse genetic studies have added valuable information to that obtained from traditional studies. However, we still have no answer to the fundamental question regarding sexual development: ‘what triggers gametocytogenesis’? In the current climate of eradication/elimination, tackling transmission by killing gametocytes has an important place on the agenda because most antimalarial drugs, whilst killing asexual blood stage parasites, have no effect on the transmissible stages.

## Graphical abstract

A stage V *Plasmodium falciparum* gametocyte in a blood film fixed with methanol and stained with Giemsa. We do not understand how differentiation to these sexual forms occurs in the host, but if it could be prevented with drugs then this would be a valuable tool to control malaria transmission.

## 1 Introduction

Malaria is caused by protozoa of the genus *Plasmodium*; these are apicomplexan parasites and are part of the alveolate grouping [1]. As well as other animal pathogens such as *Toxoplasma*, *Crytptosporidium*, *Theileria* and *Eimeria*, alveolates also comprise free living ciliate protozoa such as *Paramecium* and *Tetrahymena* and also the dinoflagellates. Asexual proliferation of malaria parasites within the red blood cells of a human host leads to the pathology and symptoms of malaria. The malaria parasite life cycle also comprises a phase of sexual reproduction which takes place in the mosquito vector, but the actual switch to sexual development occurs in the vertebrate host, with the formation of male and female gamete precursors (gametocytes), and is a prerequisite for transmission of disease [2]. This review will focus on our current knowledge of the biology of gametocytes and their development, and will highlight how the parasite genome sequence and post-genomic approaches have advanced our understanding of this important life cycle stage. The majority of the information has been derived from research carried out on the major human pathogen *Plasmodium falciparum* where the blood stages can readily be cultured *in vitro* [3], but also the rodent malaria parasite *Plasmodium berghei* which is a genetically more amenable system and provides an important *in vivo* model [4]. It is encouraging that advances in our knowledge are being made continuously, though consequently a review can provide a snapshot at best. The nature of reverse genetic techniques currently applicable to *Plasmodium* provided a window of opportunity to advance the study of sexual stage genes and has facilitated recent progress. The recent funding injection to support malaria elimination/eradication efforts and the resulting growing recognition of the importance of tackling transmission [5,6] will provide an opportunity to further increase our understanding of the biology of these fascinating life cycle stages.

## 2 The distinct morphology of gametocytes compared to the asexual blood stages is reflected in their unique pattern of gene expression

Though a large proportion of the work on *Plasmodium* gametocytes has been carried out on *P. falciparum*, the extended period (8–12 days) of development (see [6] for review) is unique to this species (and the closely related primate malaria parasite *Plasmodium reichenowi* [2]). Therefore some of the findings may not be relevant to other malaria species. *P. falciparum* gametocyte development can be conveniently divided into five morphologically recognisable stages (I–V) according to a widely used classification system [7]. This system documents the distinctive changes in appearance that occur following invasion of a red cell by a sexually committed merozoite. After the (sexually committed) ring stage, the gametocyte grows and elongates to gradually occupy the majority of the host red cell. Stage I gametocytes (up to ∼40 h post-invasion) are difficult to distinguish from young trophozoites in a Giemsa-stained blood film, but are roundish and a pointed end and distinctive pigment pattern may be visible. Specific antibodies to early gametocyte markers using IFA are required to be 100% certain of the identity of stage I gametocytes. For many years the membrane protein Pfs16 has been a valuable marker of gametocytogenesis; its expression has been measured at 24 h post-invasion [8] and persists throughout gametocyte development. Electron microscopy revealed that this protein is located in the parasitophorous vacuole membrane (pvm) as well as in the membrane surrounding ingested food vacuoles and in membranous clefts in the red cell cytoplasm. The protein is lost from the parasite during gametocyte activation as the pvm is disrupted in characteristic multilaminate whorls and makes way for the emerging gametes [9,10]. It has been noted that other early gametocyte proteins Pfpeg3 and Pfpeg 4 expressed in stage II gametocytes [11]; PF14_744 and PF14_748 [12] are also located in the pvm. The common pvm location of all these early proteins has been suggested to reflect the requirement to remodel this host-derived capsule in a manner distinct from that required in asexual blood stage parasites [13]. Another early gametocyte specific protein (Pfg27) is transcribed at ∼30 h post-invasion [14]. This is a cytoplasmic RNA-binding phosphoprotein [15] that intriguingly has no orthologue in other malaria parasite species (apart from *P. reichenowi*), leading to the hypothesis that it may have a role in the extended period of gametocyte development. Deletion of this gene allows only a fraction of gametocytes to develop normally, but most *Pfg27*^*−*^ gametocytes are morphologically abnormal [16].

Gametocyte stages II–V can all be readily distinguished in Giemsa-stained blood films with practise and their morphologies have been depicted elsewhere [13,17]. The individual stages of gametocyte development have also been described at the ultrastructural level by Sinden [18]. This EM-based study gives details of the development of the sub-pellicular microtubule-based cytoskeleton and the surrounding interrupted, double membrane which determine the characteristic shape of gametocytes. The highly distinct morphology and, to some extent, the extended period of development made it obvious that *P. falciparum* gametocytes would express a large number of proteins specific to this stage of development. It also became clear that sexual stage specific promoters were present that regulate the precise temporal expression of genes. For example, in one of the first studies to examine this issue, transfection of *Plasmodium gallinaceum* with reporter constructs containing a segment of 5′ UTR of the Pfs16 and Pfs25 genes upstream of GFP was sufficient to direct expression in a sexual stage specific manner, and also governed appropriate temporal expression [19].

Examination of the *P. falciparum* proteome detected more than 900 proteins in gametocytes; 315 of these were found exclusively in gametocytes. More than 600 proteins were found in gametes and 97 of these were gamete-specific [20]. Around 250–300 genes specifically upregulated at the mRNA level during *P. falciparum* gametocyte development have been detected by transcriptome analysis [11,21]. A sex-specific proteome study in *P. berghei* has shown that a large proportion of the 5–600 proteins are expressed exclusively in either male or female gametocytes and relatively few are expressed in both, reflecting the individual roles of the two sexes. This study utilised the fact that gametocytes have sex-specific promoters and two of these were used to drive expression of distinct fluorescent tags allowing cell sorting [22]. Proteins identified in males included those that will subsequently be involved in axoneme formation and motility, but also DNA replication that follows activation. The distribution of mitochondrial enzymes and proteins involved in protein synthesis were biased towards female gamete preparations. Sexual dimorphism is morphologically most apparent from stage IV onwards in *P. falciparum* and this stage is characterised by an elongated shape with both ends pointed. The female gametocyte or ‘macrogametocyte’ is characterised by a relatively small nucleus (with a nucleolus) and concentrated pigment pattern; by contrast the nucleus is larger (apparently lacking a nucleolus) and the pigment more diffuse in the male (‘microgametocyte’). The frequency of electron dense osmiophilic bodies at the periphery of the cytoplasm is significantly higher in female gametocytes. Secretions from osmiophilic bodies are involved in host membrane disruption during gamete emergence from red blood cells [23,24] and deletion of Pfg377 has revealed a role for this protein in osmiophilic body formation and female gamete emergence [25]. Ribosomes are also found at higher densities in female gametocytes accounting for their bluer appearance in Giemsa-stained blood films (pink in males). Stage V or mature gametocytes have rounded ends due to depolymerisation of the sub-pellicular microtubules [18] and may be sausage or crescent-shaped.

## 3 Gametocyte metabolism

### 3.1 Nucleic acid synthesis

There are intriguing earlier observations suggesting the possibility of DNA synthesis and nuclear division in *P. falciparum* and *P. berghei* gametocytes [18,26]. However, current opinion is that there is no replication of the genome in gametocytes; tritiated hypoxanthine is apparently not taken up by gametocytes [27]. It is likely that nucleic acid synthetic activity in gametocytes is restricted to RNA synthesis and that, consistent with genetic evidence, gametocytes are haploid and that rapid genome replication and nuclear division occur in male gametocytes only after activation in the mosquito midgut. In the vertebrate host, gametocytes appear to be developmentally arrested at the G_0_ phase of the cell cycle.

Synthesis of RNA is thought to occur up to around day 6 of *P. falciparum* gametocyte development and this (as well as protein synthesis and haemoglobin digestion) is reported to cease in mature gametocytes [28,29]. Experimentation with northern blots and gametocyte cDNA libraries in the late 1980s- early 1990s revealed a surprisingly high proportion of transcripts to be ‘sexual stage specific’. It was puzzling for example that so many genes encoding putative signalling enzymes (e.g. protein kinases) should be expressed in this so-called quiescent stage of the life cycle. The explanation began to emerge-that some mRNAs such as those encoding p25 and p28 [30–32] were synthesised in female gametocytes, but translation was repressed until subsequent mosquito stages of the life cycle. It also became clear that this phenomenon was likely to apply to numerous transcripts in both *P. falciparum* and *P. berghei* gametocytes [33,34]. Details of the translational repression mechanism were later revealed. Immunoprecipitation was used to show association of *P. berghei* p25 and p28 mRNA (and others) in complex with DOZI that is encoded by an orthologue of enzymes in the DDX6 family of DEAD-box RNA helicases. Their roles in other organisms include storage of key mRNAs in translationally silent ribonucleotide particles (mRNPs) and translational repression [35]. DOZI was highly upregulated in the proteome analysis of female gametocytes [22]. Deletion of the *pbdozi* gene prevented ookinete formation; development of zygotes arrested development prior to meiosis [35]. In this mutant line, a total of 370 transcripts were shown to be down regulated significantly in gametocytes (interestingly 92 transcripts increased in abundance) compared to wild type parasites using microarray analysis, including the majority of those transcripts previously hypothesised to undergo translational repression [33]. It is likely that translational repression allows stock piling in the gametocyte of mRNAs that can be rapidly translated once the parasite receives the environmental cues required to trigger gametogenesis.

A unique feature of malaria parasites is the developmentally regulated expression of the dispersed, nuclear encoded small subunit ribosomal RNA (SSU rRNA) genes [36]. There are seven of these in *P. falciparum* and four in the rodent malarias. Only A-type rRNA is expressed in asexual blood stage parasites. This form is also expressed in gametocytes but also precursors of the S-type rRNA (formerly known as the C-type) are expressed in gametocytes and are processed in subsequent mosquito stages and replace the dominant A-form rRNA population at around 6 days after fertilisation corresponding with development of sporozoites. It seems that the switch between ribosome populations coincides with the distinct environmental transitions that the parasite encounters as it progresses through the life cycle.

### 3.2 Carbohydrate metabolism and energy production

It has long been known that the malaria parasite relies on glucose for energy production via anaerobic glycolysis and that parasite derived enzymes are involved [37–40]. More recent identification of all the corresponding genes in the parasite genomes has supported the biochemical evidence [41]. Organic products (such as lactate) are generated rather than complete degradation to CO_2_ and H_2_O. A trend towards decreased expression of genes involved in glycolysis, protein biosynthesis and haemoglobin catabolism was observed in later gametocyte stages [21] likely reflecting a decrease in certain metabolic processes with increasing age and corresponding to the absence or reduced gametocytocidal activity of antimalarial drugs targeting some of these processes (see later section).

### 3.3 The mitochondrion and apicoplast

A recent study has revealed important information on the morphology of the mitochondrion (and apicoplast) in gametocytes [42]. Using a GFP tagging approach and specific antibodies, markers of the mitochondrion and apicoplast have revealed that in both cases, there is only one of each of these organelles in both male and female gametocytes. The apicoplast does not show significant morphological changes during gametocytogenesis and remains small which contrasts remarkably with the development of the mitochondrion. This consists of a large branching structure observed from stage II onwards which appeared to envelop the apicoplast with which it remains closely associated and this likely facilitates (their known) metabolic cooperation. This branched structure probably accounts for previous reports of parasites having multiple mitochondria in electron micrographs. This study also demonstrated that both the apicoplast and the mitochondrion remain in the remnants of the activated microgametocyte confirming that the male gamete lacks both and supports the evidence that these organelles are maternally inherited [43,44]. It has been reported that gametocyte mitochondria have tubular cristae [45], a key diagnostic feature of alveolates, which may have implications for function. Others have stated that the mitochondria of mammalian *Plasmodium* are initially acristate, but develop cristae later at the ookinete stage [46].

It is widely viewed that in asexual blood stage parasites, mitochondria are unable to carry out full oxidation of glucose for mitochondrial ATP synthesis and it has been suggested that the main function of the mitochondrial TCA cycle is production of succinyl Co-A for haem biosynthesis. It was also pointed out that an important TCA entry point could be via glutamate from haemoglobin degradation [47]. It is also clear that a key role of the mitochondrial electron transport chain is production of orotate to fuel cytoplasmic pyrimidine biosynthesis [47,48]. However, it is thought that other dehydrogenases in the mitochondria are likely to play additional essential metabolic roles in the malaria parasite [49]. In a study examining transcripts present at the various stages of gametocyte development 15 of the 16 mRNAs encoding enzymes of the mitochondrial TCA cycle were upregulated in gametocytes [21] and nine of these were detectable at the protein level in female *P. berghei* gametocytes and five in male gametocytes in a sex-specific proteome study [22]. The latter sex-specific proportion may have functional significance. Expression of these genes and potential enzyme activity is consistent with the presence of a spectacularly expanding mitochondrion in developing *P. falciparum* gametocytes [42]. Clearly there is a requirement for a careful biochemical/genetic examination of mitochondrial function in gametocytes and mosquito stages. It will also be important to determine whether functionality varies between malaria parasite species.

There is evidence that intermediates in haem biosynthesis are transferred between the mitochondrion and the apicoplast [50]. Interestingly mRNAs corresponding to three of the six genes implicated in the haem biosynthesis pathway were upregulated during gametocytogenesis as well as six members of the type II fatty acid biosynthesis pathway [21]. In the *P. berghei* sex-specific proteome study [22] it is interesting to note that apicoplast proteins are expressed at equivalent levels in males and females indicating that these proteins are likely to be active in the male gametocyte itself rather than solely in readiness for later development (since the apicoplast is absent in the male gamete).

## 4 What causes gametocytogenesis?

It is likely that progression through the complex series of stages of the malaria parasite life cycle involves an intricate interplay between the parasite's changing environment and its own in-built genetic programme. Some of the life cycle transitions coincide with extreme changes in environment that are easy to envisage causing dramatic changes in patterns of gene expression. Examples include when a gametocyte is transported from the bloodstream into the mosquito midgut, or the sporozoite as it leaves the mosquito salivary glands and enters the mammalian bloodstream. Changes in temperature, pH, salt composition and the presence of e.g. organic chemical signals will be sensed, transduced and patterns of biochemical activity changed rapidly. However, one dramatic life cycle transition that does not require an abrupt change of environment (due to the absence of a change in location or host) is the ‘decision’ to trigger the genesis of gametocytes.

Numerous papers have described manipulation of culture conditions and addition of pharmacological agents which can increase gametocyte numbers or apparently initiate gametocytogenesis. For example, addition of red cell lysate has been shown to increase the rate of gametocytogenesis [51] as has the presence of human serum and lymphocytes [52], mammalian hormones [53,54], high levels of reticulocytes [55], some inhibitors of nucleic acid synthesis [56] including antifolates [57] and also chloroquine in [58–60]. Furthermore it has also been reported that apparent gametocyte non-producers can be stimulated to produce gametocytes by addition of Berenil and ammonia compounds [56,61]. It has been suggested that haematological disruption, i.e. red cell lysis, anaemia, dyserythropoiesis and reticulocytosis is the key to changes that are linked to gametocyte production [62]. There is evidence that the appearance of gametocytes in *P. falciparum* cultures correlates with high asexual parasite density [63]. It has also been shown that diffusible factors in cultures can favour gametocytogenesis [64]. However, it has further been suggested that diffusible factors from asexual blood stage parasites in log phase may repress gametocytogenesis, but when parasitaemia reaches a critical threshold, this repression is lifted. It was postulated that gametocytogenesis might actually be the default developmental pathway which is reminiscent of the fact that all merozoites of haemoproteids (apicomplexan relatives), following invasion of avian red blood cells, become gametocytes by default [6]. In members of the more distant alveolate ciliate relatives, helical protein ‘pheromones’ are secreted that interact with other cells to cause growth arrest and they become sexual forms [65].

There are also reports of potential signalling pathways that may be involved in triggering gametocytogenesis. For example, phorbol ester inducing pathways [66] and the cAMP signalling pathway [66–70]. An intriguing increase in conversion to sexual development was observed on addition of cholera toxin to *P. falciparum* cultures [71]. The conclusion was that a G protein-dependent signalling system may mediate the switch to sexual development in response to environmental factors. The apparent identification of heterotrimeric G_α_ subunits in this study is puzzling due to the lack of obvious encoding genes in the *Plasmodium* genome data. Whether the later report of involvement of host red blood cell G protein signalling in *P. falciparum* infection can reconcile these observations remains to be seen [72]. Interestingly, the *Plasmodium* genomes encode potential homologues of G protein-coupled receptors from the plant, *Arabidopsis thaliana*, that have been shown to have both intrinsic GDP/GTP exchange and GTPase activities; thus potentially functioning as ‘hybrid’ G protein-coupled receptors/Gα subunits [73] (Joanne Thompson, personal communication).

However, none of these studies have given conclusive insight into the physiological mechanism by which the parasite switches to sexual development. It is possible that there may not be a single mechanism and that multiple factors may contribute to the decision to form gametocytes. A thought provoking model for switching to sexual development has been proposed which requires that individual parasites vary in their sensitivity to the environment [74]. Sexual commitment is also viewed by some as a stress response that allows the parasite to escape from an increasingly unfavourable environment. However, an alternative view is that the stress associated with gametocyte production may not be causal. This view is based on the evidence that in at least some *P. falciparum* infections, gametocytogenesis can be triggered at the onset of infection prior to the onset of symptoms [75]. So far *in vivo* studies in humans have not helped to solve the problem of what triggers gametocytogenesis because it is not possible to predict when or if gametocytes will develop during the course of a *P. falciparum* infection; this varies between individual patients and many host factors could potentially influence the rate of conversion of asexual parasites to gametocytes [17]. The study of gametocytogenesis *in vivo* is also complicated by the fact that the developing stages sequester and only mature forms are present in the peripheral circulation. The stage of an infection at which gametocytes first appear, contrasts between *P. falciparum* and *Plasmodium vivax*. Gametocytes can appear simultaneously with asexual parasitaemia in *P. vivax* perhaps indicating that liver stage schizonts as their source. In *P. falciparum* infections, the appearance of gametocytes is usually later and more sustained. However, the relatively late appearance is expected since the maturation period of gametocytes is much longer for *P. falciparum*.

Discovery of the molecular mechanism that triggers gametocytogenesis is arguably the ‘Holy Grail’ of malaria parasite biology. Once this is achieved it may simultaneously give insight into the secrets of sex determination in the parasites. When pulse field gel electrophoresis became available to physically separate chromosomes, the possibility of sex chromosomes was explored, but it became clear that these do not exist in malaria parasites and that gametocyte-specific and sex-specific genes are dispersed amongst the 14 chromosomes. It has also been clear for many years that all the necessary information to form both female and male gametocytes is carried within a single cloned asexual blood stage parasite. In *P. falciparum* the decision to become a gametocyte appears to be made in the asexual cycle prior to the one where gametocytes are formed. Current *in vitro* evidence supports the view that a *P. falciparum* merozoite is pre-committed to sexual development before egress from the schizont. As a result, it appears that all the merozoites derived from a single schizont, will become either gametocytes or asexual trophozoites [63] and furthermore evidence suggests that all the merozoites from a sexually committed schizont will become either male or female [76,77]. It has been shown using plasmids expressing GFP under the control of sexual stage specific promoters (PF14_0748 [12]; Pfs16 (C. Swales and D. Baker, unpublished data); Pfg27 (A. Olivieri and P. Alano, unpublished data)) that these promoters are active in a sub-population of segmented schizonts the Pfs16 promoter. This supports the view that the sexual switch has taken place prior to merozoite release in the asexual cycle preceding gametocyte formation.

## 5 *P. falciparum* isolates vary in their capacity to produce gametocytes in the laboratory

It is clear that some laboratory adapted isolates can readily produce gametocytes *in vitro* and others cannot. Clone 3D7 and its parental isolate NF54 are very reliable and widely used gametocyte producer lines. However, they lose the ability to produce gametocytes with increasing *in vitro* passage number [78] suggesting that parasites that undergo only asexual division are selected and that these might have lost genes necessary for sexual development. It was reported that *P. falciparum* isolates with a short form of chromosome 9 (using Southern analysis of pulsed field gel separations) had lost [79] (or greatly reduced [80]) the ability to produce gametocytes (and concomitantly cytoadherence) suggesting that a gene or genes in the absent segment of the chromosome might be involved in gametocytogenesis (and cytoadherence). Interestingly, selection of adherent parasites on myeloma cells (expressing CD36), also restored the ability to produce gametocytes (by a factor of 10) and these parasites had a full length chromosome 9 [81]. Subsequent work analysed genes in this region of chromosome 9 including *Pfgig* [82]. Transformants in which *Pfgig* had been disrupted showed a reduced ability (∼5-fold) to produce gametocytes. Whether this sheds light on the mechanism of gametocytogenesis or blockage of one step during gametocyte development is not yet known. The gradual loss of the ability of *P. falciparum* to produce gametocytes in culture over time makes it very difficult to identify genes that are involved in gametocytogenesis using a gene knockout approach due to the extended period of drug selection required. Therefore complementation experiments that reverse the ‘inability to produce gametocyte phenotype’ by reintroduction of the gene into the actual knockout line are required. Recently it has been shown that complementation of a gametocyte-deficient parasite line that has a 19 kb deletion in this region of chromosome 9 with an open reading frame, designated Pfgyi1 and previously known as cytoadherence-linked protein normally found in this segment, rescues the phenotype [83]. It is therefore clear that expression of this gene is required for gametocyte production. The ability to retain gametocyte production in *P. falciparum* lines by selection of cytoadherent parasites (to reduce loss of chromosome ends) on e.g. Gelofusin et al. [84] is both an interesting observation in this context and a very useful tool. It is also clear that laboratory adapted lines exist in which gametocytogenesis is blocked at different stages. For example, clone K1 and clone T996 have been described as ‘gametocyte non-producers’. Examination of these clones by IFA with a monoclonal antibody to Pfs16 (an early marker of gametocytogenesis) shows that stage I gametocytes are quite abundant, but are not apparent by Giemsa staining (unpublished data). In these lines, the ‘block’ has occurred sometime after the initial switch to sexual development. Other truly gametocyte non-producer lines have been produced in *P. falciparum* [23] and *P. berghei* [85] and have proved very useful tools for investigating gametocyte biology.

It is possible that the inability to produce gametocytes *in vitro* may not be due to deletion of one or more genes, but to mutation of key genes or promoter regions that influence the actual switch to sexual differentiation or its progression. Alternatively, an epigenetic mechanism might be involved in regulation of gametocytogenesis that is similar to that demonstrated for the mutually exclusive expression of subtelomeric *var* genes [86,87]. Here inactive *var* genes are located at the nuclear periphery and silenced by association with PfSir2. Derepression of transcription occurs following exit from the PfSir2 cluster. It is possible that if genes involved in the switch to gametocytogenesis are located in subtelomeric regions, then they may be regulated in a similar way. A defect in this epigenetic regulatory mechanism might explain why isolates lacking deletions cannot produce gametocytes (Cathy Taylor, PhD thesis, 2007). A recent study using a histone deacetylase inhibitor in combination with global transcriptional analysis strongly suggests that epigenetics plays an important role in stage specific gene expression in *P. falciparum* [88].

## 6 Sex ratios

Although a female gametocyte gives rise to a single gamete and a male gametocyte produces eight gametes following gametogenesis in the mosquito, the sex ratio of gametocytes in the vertebrate host is very variable. Sex ratio tends to be female biased in all *Plasmodium* species (reviewed by Carter and Graves) [17]. It has been reported that the sex ratio of *P. falciparum* gametocytes varies significantly between individual gametocyte carriers and over the course of infection. In a study in Cameroon the mean proportion of male to female gametocytes was 0.217 (that is 3.6 females to each male) [89]. However, the mechanism by which sex ratios are regulated has been the subject of much research and debate. Sex ratios are thought to vary in response to environmental factors that influence fertility and also in response to the number of genotypes in an infection and that this ratio affects transmission success [90]. Conditions which adversely affect male fertility redress the balance favouring male production. Induction of erythropoesis in the host has been shown to increase the proportion of male gametocytes produced in *P. gallinaceum* suggesting that host hormones can influence sex ratios [91]. Recent experimental data have indicated that rodent malaria parasites, like multicellular organisms, ‘obey the rules’ of the sex allocation theory and sex ratio is an important determinant of fitness in *P. chabaudi*. Parasites appear to be able to detect the presence of both identical and co-infecting genotypes and to vary their sex ratio accordingly during the course of infection; in multiple infections the sex ratio tends towards 1:1 [92]. It will be extremely interesting to determine which parasite molecules monitor environmental conditions and recognition of non-identical phenotypes in order to influence sex determination.

## 7 Gametocyte proteins with a subsequent role in fertilisation

The peripheral membranes of a gametocyte comprise the outermost red blood cell membrane, the pvm and the plasma membrane. The gametocyte also has a pair of incomplete membranes beneath the plasma membrane surrounding the pellicle. This double membrane is absent from all asexual blood stage parasites apart from merozoites. A number of proteins have been characterised that are located on these various membranes. Pfs48/45 and Pfs230 are located as a complex on the plasma membrane of the gametocyte (see [93] for review) with only Pfs48/45 being directly anchored to the membrane by a GPI moiety. Following activation of gametocytes in the mosquito midgut, these proteins become directly exposed to the blood meal on the extracellular male and female gamete. It is thought they play a crucial role in fertilisation. Monoclonal antibodies raised to these proteins are able to block transmission when fed to mosquitoes using an artificial membrane feeding apparatus and they have long been viewed as promising transmission blocking vaccine candidates (see [94] for review). Whilst there is a high degree of conservation of the protein sequences between diverse isolates, there are a small number of substitutions in geographically distinct populations [95]. Deletion of the gene encoding P48/45 in both *P. berghei* and *P. falciparum* has demonstrated that the protein is an important determinant of male fertility [96]. Deletion of Pfs230 has revealed a role for this protein in the characteristic agglutination of extracellular gametes with uninfected red blood cells observed during exflagellation. Pfs230^−^ mutant males develop normally, but are unable to bind to red cells within centres of exflagellation following gametocyte activation, resulting in a significant reduction in mosquito infection after membrane feeding [97]. There are also a group of six adhesive proteins (PfCCP family [98] or Lap in *P. berghei* [99]) that are expressed in the parasitophorous vacuole, but are later secreted and all six members seem to form an extracellular multi-protein complex that is important for cell–cell interaction of newly emerged female *P. falciparum* gametes. Interestingly one of these proteins PfCCp4 has been shown to associate with Pfs230 [100]. The CCP proteins are hypothesised to play a role in mediating signalling events during fertilisation as well as having subsequent roles during development within the mosquito or in the initial gamete–gamete binding event [98]. Interestingly a homologue of HAP2, responsible for a non-fusion phenotype in gametes of the single cell alga *Chlamydomonas*, was shown to be essential for fertilisation (but not initial gamete binding) in *P. berghei* [101]. It is clear that some of the important players in fertilisation of *Plasmodium* gametes have being identified as well as insight gained regarding their respective roles.

## 8 Cytoadherence and sequestration

The parasitophorous vacuole and surrounding pvm are significant barriers to be overcome by proteins that are exported to the red cell cytoplasm and beyond to the red cell surface. It has been shown that proteins containing a short conserved sequence motif (referred to as Pexel or HT) near to the N-terminus are able to traverse the pvm [102,103] in both sexual and asexual parasites. This has been particularly well studied for the family of *var* gene products (PfEMP1) involved in cytoadherence of asexual blood stage parasites to endothelial cells during sequestration of parasitised red cells within the microvasculature. Little is known about protein trafficking and cytoadherence in gametocytes. However, it is known that developing gametocytes also sequester *in vivo*. Only mature stage V gametocytes (and probably also sexually committed ring stage parasites) appear in the circulation. Early reports indicated that the spleen and bone marrow are the predominant sites occupied by developing gametocytes [104]. It has been shown that a particular *var* gene subset (type C) is transcribed in gametocytes *in vitro* and that the pattern of expression is independent of the PfEMP1 phenotype of the asexual parasites from which the gametocytes were derived [105]. Up to 40–48 h of development, gametocytes were found to cytoadhere to endothelial cells expressing CD36 via PfEMP1 in a similar fashion to asexual parasites [106]. Later stage gametocytes have been shown adhere to cells expressing ICAM-1 and binding was released by antibodies to three host cell receptors present in human bone marrow and stromal cell lines [107]. The mechanisms underlying clonal antigenic variation of *var* gene products in asexual parasites and their role in host immune evasion is well documented elsewhere. Another subtelomeric multigene family *stevor* has been hypothesised to have a role in gametocyte cytoadherence [108]. Subsets of this family are expressed in gametocytes and in contrast to *var* gene expression, the same *stevor* subset is expressed in the asexual parasites from which they are derived [105].

Reports on the immune response to host red blood cell surface-localised gametocyte proteins are more limited, but it has been demonstrated using flow cytometry that antibodies are present in Gambian children (gametocyte carriers 1–2 weeks following drug treatment) that react with antigens exposed at the infected red cell surface of cultured mature *P. falciparum* (clone) 3D7 gametocytes. These were distinct from those expressed at the red cell surface of asexual blood stage 3D7 parasites and may have a role in modulating transmission since the presence of these antibodies between days 7 and 14 correlated with reduced gametocyte densities at day 28 following drug treatment [109]. Study of these red cell components during gametocyte development and their interaction with host tissues is an important area for future research and may reveal novel means to control transmission. It has also been hypothesised recently that proteins expressed on the red cell surface of gametocytes may play a role in determining when mature gametocytes are released into the circulation and moreover where they go to maximise their chances of being picked up by a mosquito [110].

## 9 Drugs that affect gametocyte development and reduce transmission

It could be argued that the ideal antimalarial drug should kill both asexual blood stage parasites and gametocytes, thus curing the individual and protecting the population by preventing onward transmission of infection. There are also arguments favouring the combined use of distinct drugs (that inhibit distinct molecular targets) to kill these two individual life cycle phases to reduce the spread of drug resistance. The point has also been well made that the mosquito stages of development are vulnerable in terms of the extremely low parasite numbers and are thus an excellent target for drugs (or vaccines) [6]. There is a great deal of early work showing that treatment of patients with antimalarial drugs inhibits subsequent parasite development effectively (e.g. inhibitors of dihydrofolate reductase such as pyrimethamine) in the mosquito at the various stages of development following a blood meal (sporontocidal drugs, reviewed by Butcher [59]). However, persuading funders to support translational research that focuses on transmission-specific drug targets is understandably challenging. With the recent availability of funding to attempt elimination/eradication of malaria, there is a growing recognition of the importance of controlling transmission [5]. Screening for new compounds that are active against developing and mature gametocytes is underway in several locations worldwide.

Most antimalarial schizonticides have no effect on gametocyte development and therefore whilst curing the patient, allow transmission of disease to others in the population for weeks after clearance of asexual parasites [111]. Furthermore there is evidence of enhanced rates of transmission of drug resistant parasites from drug-treated individuals [112]. Other drugs such as quinine and mepacrine [113] have even been reported to increase gametocyte numbers or transmission to mosquitoes. Some drugs such as chloroquine (up to around day 6 of development) [114] and quinine kill early gametocytes due to their effects on degradation of haemoglobin, but have no effect on the later stages. Atovaquone is known to selectively inhibit the parasite ubiquinol oxidation site (*Q*_0_) of cytochrome *b* that is involved in the maintenance of mitochondrial membrane potential [115–117] and is active against the younger gametocyte stages [118]. The favourable effects of artemisinin derivative-based combination therapy (ACT) on transmission are also well documented [119]. Although artemisinin derivatives can reduce gametocyte carriage [120,121] transmission can still occur following ACT [122] which may in part reflect the rapid clearance of artemisinin-based compounds. It is likely that the reduction in gametocyte numbers following ACT at least partly reflects an indirect effect due to the rapid clearance of asexual parasites [123]. Evidence suggests that artemisinin kills early (sequestered) forms, but not mature gametocytes [121,124–127], but more detailed *in vitro* studies on this would be worthwhile.

Primaquine (used widely to treat *P. vivax* and *P. ovale* infection) and other 8-aminoquinolines can certainly kill mature *P. falciparum* gametocytes [59,128–130]. Prior to the introduction of ACT as a first line treatment, a single dose of primaquine was recommended by WHO to reduce transmission in low endemic areas [131]. 8-Aminoquinolines have also been included previously in mass drug administration programmes [132]. A single dose of primaquine has been used successfully in children in a hypedrendemic region of Tanzania to clear residual gametocytes following treatment with sulphadoxin-pyrimethamine and artesunate [127]. It was suggested that regimes including a single dose of primaquine could be used to reduce post-treatment infectivity and importantly reduce the likelihood of transmission of drug resistant parasites. The main problem with primaquine is a significant safety issue especially in areas with high levels of glucose-6 phosphate dehydrogenase deficiency where serious haemolytic anaemia can occur in some forms of the condition (reviewed by Vale et al. [130]). The seemingly unique ability of primaquine and its relatives to kill mature *P. falciparum* gametocytes (as well as hypnozoites of *P. vivax* and *P. ovale*) necessitates discovery of its molecular target which, since 8-aminoquinolines have a relatively poor activity against asexual parasites, is likely to be a component of one of the biochemical pathways that are specific to gametocytes (and hypnozoites). Though its mode of action is not known, there is evidence that it might target mitochondrial function (reviewed by Vale et al. [130]) and if so this is likely to be some aspect that is of particular importance to maturing gametocytes.

## 10 Concluding remarks

Though the study of gametocytes has received relatively little attention in the past compared to their asexual blood stage counterparts, there currently appears to be renewed vigour in the field. As a new generation of scientists turn their attention to this fascinating life cycle stage and apply the ever improving new technologies, I am confident that during the next decade, gametocytes will be forced to reveal some of their most important secrets. I apologise to those whose relevant work has been omitted due to space constraints.
